# Dynamic culture of human liver equivalents inside a micro-bioreactor for long-term substance testing

**DOI:** 10.1186/1753-6561-7-S6-P72

**Published:** 2013-12-04

**Authors:** Eva-Maria Materne, Ilka Wagner, Caroline Frädrich, Ute Süßbier, Reyk Horland, Silke Hoffmann, Sven Brincker, Alexandra Lorenz, Matthias Gruchow, Frank Sonntag, Udo Klotzbach, Roland Lauster, Uwe Marx

**Affiliations:** 1 TU Berlin, Institute for Biotechnology, Faculty of Process Science and Engineering, Gustav-Meyer-Allee 25, 13355 Berlin, Germany; 2 Fraunhofer IWS Dresden, Winterbergstraße 28, 01277 Dresden, Germany; 3 TissUse GmbH, Markgrafenstraße 18, 15528 Spreenhagen, Germany

## Background

Current *in vitro *and animal tests for drug development are failing to emulate the organ complexity of the human body and, therefore, to accurately predict drug toxicity. In this study, we present a self-contained, bioreactor based human *in vitro *tissue culture test system aiming to support predictive substance testing at relevant throughput. We designed a microcirculation system interconnecting several tissue culture spaces within a PDMS-embedded microfluidic channel circuit. The bioreactor is reproducibly perfused by a peristaltic on-chip micro-pump, providing a near physiologic fluid flow and volume to liquid ratio.

## Materials and methods

Liver microtissue aggregates containing 4.8 × 10^4 ^HepaRG cells and 0.2 × 10^4 ^human hepatic stellate cells (HHSteC) were formed in Perfecta3D^® ^384-Well Hanging Drop Plates (3D Biomatrix, USA). After two days of hanging drop culture, 20 aggregates were loaded into a single tissue culture compartment of the micro-bioreactor. Each circuit of the micro-bioreactor device contained 700 μl medium in total. During the first 7 days, a 40% media exchange rate was applied at 12 h intervals. From day 8 onwards, a 40% exchange rate was applied at 24 h intervals. Daily samples were collected for respective analyses. Experiments were stopped at day 14 and 28 and tissues were subjected to immunohistochemical stainings and qRT-PCR analyses. Experiments were conducted with four replicates. To expose the chip-cultures to troglitazone, liver microtissues were cultured for one day in normal medium and were, subsequently, treated with 0 μM, 5 μM and 50 μM substance, respectively. Application of troglitazone was repeated at 12 h or 24 h intervals simultaneously with the medium change.

## Results

**Figure 1:**
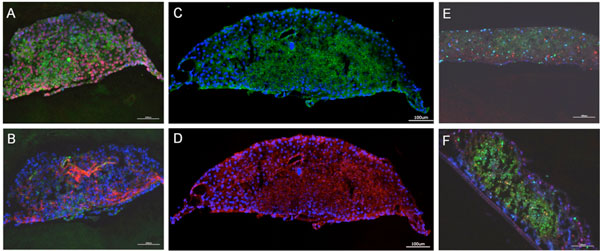
**14-day tissue performance of the micro-bioreactor culture compared to static control Cell functionality shown by immunostaining of **(A) **phase I enzyme CYP450 3A4 (red) and CYP450 7A1 (green), **(B) **collagen I (red) and vimentin (green), **(C) **MRP2, an ABC transporter located at the apical membrane, (green) and **(D) **tight junction protein ZO-1 (red)**. Cell viability shown by TUNEL KI67 staining of **(E) **liver equivalents cultivated for 28 days in the micro-bioreactor and **(F) **liver equivalents cultivated for 28 days under static conditions. Nuclei are stained with hoechst 33342. Scale bar: 100 μm.

Cultures of human artificial liver microtissues have successfully been cultivated over 28 days in the novel microfluidic bioreactor. Glucose consumption and lactate production indicated an aerobic metabolism which reached a steady state after 7 days. Immunohistochemical staining revealed the expression of phase I metabolic enzymes CYP450 3A4 and CYP450 7A1, extracellular matrix component collagen I, apical transporter MRP2 and tight junction protein ZO-1 (Figure 1A-D). Cell viability over 28 days was increased in the bioreactor culture compared to static control (Figure 1E, F). Furthermore, the cultures revealed a dose-dependent response to a 7-day exposure to the toxic substance troglitazone. Liver microtissues showed sensitivity at different molecular levels. Concentration of LDH released to the medium increased with troglitazone concentration and gene expression of selected marker genes varied. An induction of CYP450 3A4 by troglitazone treatment was also recorded on protein level by immunhistochemistry.

## Conclusion

A promising tool for long term culture of human liver equivalents has been developed. The simple MOC design presented, assisted the culture of human liver equivalents over a period of up to 28 days. The cultures, operated at a total on-chip volume of 700 μl medium at recirculation rates of 40 μl/min assisted by an on-chip micropump, stabilize approximately within a week at a metabolic steady state. The prediction of toxicology profiles of compounds metabolised by the liver was demonstrated possible by exposing the cells to different concentrations of troglitazone. This platform is designed to generate high-quality in vitro data predictive of substance safety in humans. Tissue cultures can be exposed to pharmaceutical substances at regimens relevant to respective guidelines, currently used for subsystemic substance testing in animals.

